# Gene Combination Transfer to Block Autoimmune Damage in Transplanted Islets of Langerhans

**DOI:** 10.1080/15438600490486822

**Published:** 2004

**Authors:** Suzanne Bertera, Angela M. Alexander, Megan L. Crawford, Glenn Papworth, Simon C. Watkins, Paul D. Robbins, Massimo Trucco

**Affiliations:** 1 Division of Immunogenetics Department of Pediatrics University of Pittsburgh School of Medicine Rangos Research Center, Children's Hospital of Pittsburgh, 3460 Fifth Avenue Pittsburgh Pennsylvania 15213 USA; 2 Department of Cell Biology and Physiology University of Pittsburgh Pittsburgh Pennsylvania USA; 3 Department of Molecular Genetics and Biochemistry University of Pittsburgh School of Medicine Pittsburgh Pennsylvania USA

## Abstract

Islet transplantation therapy would be applicable to a wider range of diabetic patients if donor islet acceptance and protection were possible without systemic immunosuppression of the recipient. To this aim, gene transfer to isolated donor islets ex vivo is one method that has shown promise. This study examines the combined effect of selected immunomodulatory and anti-inflammatory genes known to extend the functional viability of pancreatic islet grafts in an autoimmune system. These genes, indoleamine 2,3-dioxygenase (IDO), manganese superoxide dismutase (MnSOD), and interleukin (IL)-1 receptor antagonist protein (IRAP), were transferred to isolated NOD donor islets ex vivo then transplanted to NOD*scid* recipients and evaluated in vivo after diabetogenic T-cell challenge. The length of time the recipient remained euglycemic was used to measure the ability of the transgenes to protect the graft from autoimmune destruction. Although the results of these cotransfections gave little evidence of a synergistic relationship, they were useful to show that gene combinations can be used to more efficiently protect islet grafts from diabetogenic T cells.


*Experimental Diab. Res.*, 5:201–210, 2004
Copyright © Taylor and Francis Inc.
ISSN: 1543-8600 print / 1543-8619 online
DOI: 10.1080/15438600490486822

Taylor & Francis
Taylor & Francis Group

### Gene Combination Transfer to Block Autoimmune Damage in Transplanted Islets of Langerhans

**Suzanne Bertera,[1] Angela M. Alexander,[1] Megan L. Crawford,[1] Glenn Papworth,[2] Simon C. Watkins,[2] Paul D. Robbins,[3] and Massimo Trucco[1]**

[1]*Division of Immunogenetics, Department of Pediatrics, University of Pittsburgh School of Medicine, Rangos Research Center, Children's Hospital of Pittsburgh, Pittsburgh, Pennsylvania, USA*
[2]*Department of Cell Biology and Physiology, University of Pittsburgh, Pittsburgh, Pennsylvania, USA*
[3]*Department of Molecular Genetics and Biochemistry, University of Pittsburgh School of Medicine, Pittsburgh, Pennsylvania, USA*

**Islet transplantation therapy would be applicable to a wider range of diabetic patients if donor islet acceptance and protection were possible without systemic immunosuppression of the recipient. To this aim, gene transfer to isolated donor islets ex vivo is one method that has shown promise. This study examines the combined effect of selected immunomodulatory and anti-inflammatory genes known to extend the functional viability of pancreatic islet grafts in an autoimmune system. These genes, indoleamine 2,3-dioxygenase (IDO), manganese superoxide dismutase (MnSOD), and interleukin (IL)-1 receptor antagonist protein (IRAP), were transferred to isolated NOD donor islets ex vivo then transplanted to NOD*scid* recipients and evaluated in vivo after diabetogenic T-cell challenge. The length of time the recipient remained euglycemic was used to measure the ability of the transgenes to protect the graft from autoimmune destruction. Although the results of these cotransfections gave little evidence of a synergistic relationship, they were useful to show that gene combinations can be used to more efficiently protect islet grafts from diabetogenic T cells.**

**Keywords** Adenoviral Vector; Combination Gene Therapy; IDO; IRAP; Islet Transplantation; MnSOD; Type 1 Diabetes

Type 1 diabetes is a disease that results from the T-cell–mediated progressive and selective destruction of pancreatic beta cells. These cells, located in the islets of Langerhans, physiologically produce insulin as a regulatory response to glucose levels in the blood. Transplantation of donor islets is gaining favor as an alternative therapy to multiple, daily insulin injections for treatment of this disease. The recent success of an islet transplantation regimen developed in Edmonton, Alberta, Canada, has led to long-term euglycemia for some diabetic patients [1]. However, the price paid for this cure is the life-long administration of systemic immunosuppressive drugs. Even though these drugs are nonsteroidal and therefore less toxic for the beta cells themselves, this long-term therapy is only recommended for patients who have "brittle" diabetes and are prone to failure in managing hyperglycemia sufficiently well with exogenous insulin. Therefore, this therapy is not recommended for young, type 1 diabetic patients who are able to manage their disease with daily injections of insulin. In these cases, the risks of life-long insulin therapy are preferred to the risks of drug toxicity from a life-long immunosuppressive regimen [2].

One of the ways being explored to improve islet replacement therapy is to manipulate donor islets before transplantation by

Received 23 October 2003; accepted 4 April 2004.
This work was supported in part by the Juvenile Diabetes Research Foundation grant no. 4-1999-845. The MnSOD antibody used in this study was recommended and developed by Dr. Larry Oberley of the University of Iowa. The authors wish to thank Robert Lakomy and Alexis Styche for their excellent technical assistance with flow cytometric analysis, Sean Alber for fluorescent histological staining, and Dr. Nick Giannoukakis for the ELISA results.
Address correspondence to Suzanne Bertera, PhD, Children's Hospital of Pittsburgh, Rangos Research Center, 3460 Fifth Avenue, Pittsburgh, PA, 15213, USA. E-mail: subst5@pitt.edu ex vivo gene transfer to protect the graft from destruction and circumvent the need for systemic immunosuppression of the recipient. Once this method of islet protection is successful, transplantation may benefit a wider range of diabetic patients. However, because the etiopathogenesis of diabetes is quite complex, it is unlikely that just one gene will be found that could block autoimmune insulitis, prevent islet cell dysfunction and apoptosis, protect beta cells from reactive oxygen species (ROS), and inhibit the immune rejection of islet allografts. In previous studies, we have tested some promising immunoregulatory and antioxidant genes in vitro [3–5] and in vivo, individually [3, 4]. Gene transfer was accomplished using replication-deficient, E1- and E3-deleted, first-generation adenoviral vectors to ferry the transgene into the target cells. The adeno-constructs were able to infect the outermost cells comprising each islet. The resulting expression of the cytomegalovirus (CMV) promotor–driven transgene in these outer cell layers provided enough protection from the immediate environment to extend (compared to controls) the functional life of the transplanted islets after exposure to activated diabetogenic T-cells. With the promising results of these single-gene transfers, we went further by selectively combining these genes to test whether a synergistic effect may even further prolong islet function after diabetogenic T-cell exposure. The genes we have included are indoleamine 2,3-dioxygenase (IDO), interleukin (IL)-1 receptor antagonist protein (IRAP or IL-1Ra), and manganese superoxide dismutase (MnSOD).

IDO is an enzyme involved in the catabolism of tryptophan and has been shown to play a role in the immune response by limiting T-cell proliferation [6, 7]. IRAP is a naturally occurring, nonsignaling competitor for the IL-1 receptor [8]. IRAP binding is thought to block insulitis and autoreactivity by blocking IL-1$\beta$–induced expression of inducible nitric oxide synthase (iNOS) [9] and the Fas receptor [10]. An antioxidant enzyme was included because pancreatic islets have been observed to be sensitive to oxidative damage [11]. MnSOD in particular was chosen because it is expressed in response to inflammatory cytokines [12, 13] but is not found in abundance in pancreatic islets [14]. Three combinations of these genes, IDO plus MnSOD, MnSOD plus IRAP, and IDO plus IRAP, were transferred to isolated islets ex vivo by adenoviral infection.

### MATERIALS AND METHODS

### Experimental Animals

NOD/LtSzJ (NOD) female mice, 3 to 4 weeks of age (nondiabetic islet donors) or 12 to 20 weeks of age (diabetic spleen cell donors), and NOD.CB17-*Prkdc*$^{scid}$/J (NOD*scid*) female mice, 8 to 12 weeks of age (islet recipients), were obtained from Jackson Laboratories (Bar Harbor, ME). All mice were kept in an American Association of Laboratory Animal Science (AALAS)-certified animal facility in the Rangos Research Center at the Children's Hospital of Pittsburgh (CHP) and housed in microisolator caging within National Institutes of Health guidelines for animal care in a specific pathogen–free (SPF) environment. The research protocols for this experiment were approved by the CHP Animal Research and Care Committee.

### Islet Isolation and Culture

Mouse islets were isolated from the exocrine pancreas by collagenase digestion under sterile conditions, following the previously published classical method [15]. After separation on a Ficoll gradient, the islets were further purified by hand picking to eliminate any remaining exocrine tissue. Whole islets were maintained in culture medium consisting of RPMI 1640 medium supplemented with 10% fetal calf serum (FCS), 20 mM HEPES, 1% L-glutamine, 1% penicillin/streptomycin, and 50 $\mu$M $\beta$-mercaptoethanol (2-ME) at 37°C in an atmosphere of 95% air, 5% $CO_2$. Tissue culture reagents were purchased from Life Technologies (Grand Island, NY). Collagenase type V, Ficoll type 400, and 2-ME were purchased from Sigma (St. Louis, MO).

### Adenoviral Vectors

Replication-deficient, E1- and E3-deleted, first-generation recombinant adenoviral vectors were utilized in these experiments. All transgenic vectors contained the CMV promotor. The murine IDO vector was cloned into a pBluescript vector and inserted into the pQBI-AdCMV5-BFP transfer vector to produce Ad-IDO [3]. Human MnSOD was cloned into pAD·CMV link then inserted into the Ad5.sub360 transfer vector to produce Ad-MnSOD [4]. The human IRAP vector (Ad-IRAP), provided by Amgen (Boulder, CO), and the beta-galactosidase vector (Ad-LacZ), provided by Genvec (Rockville, MD), were propagated and provided by the University of Pittsburgh Vector Core [16].

### Gene Transfer to Islets

Islets were incubated singly or with combinations of Ad-IDO-BFP, Ad-IRAP, Ad-MnSOD, and Ad-LacZ vectors in serum-free medium at a multiplicity of infection (MOI) of 100 for each gene for approximately 1 hour at room temperature with agitation every 15 minutes. An MOI of 100 was chosen based on dose-response experiments showing an infection rate of 70% to 90% of the surface area of the islets without cytotoxic effects [3, 4, 17]. After infection, islets were placed back in complete media and incubated for 2 to 4 days before further analysis.

### Cell Preparation and FACS Analysis

Two to 4 days after infection with a combination of Ad-IDO-BFP and Ad-MnSOD transgenes, islets were dissociated to single cells with enzyme-free dissociation buffer (Gibco-BRL), washed, and placed in fluorescence-activated cell sorting (FACS) medium (Hank's buffered saline solution [HBSS] with 0.1% sodium azide and 0.5% bovine serum albumin [BSA]). The single cells were incubated with an MnSOD antibody for 20 minutes, then washed and fixed with 2% paraformaldehyde (PFA). Flow cytometry was done on a FACSCalibur flow cytometer (Becton Dickinson). Analysis was performed 3 times on groups of 200 islets isolated and infected on 3 separate occasions.

### Antibody Staining of MnSOD

Whole islets infected with Ad-IDO-BFP and Ad-MnSOD were fixed in a 2% PFA and permeabilized with 0.1% Triton-X solution. After blocking with goat serum, the primary rabbit polyclonal immunoglobulin G (IgG) MnSOD antibody (Upstate Biotechnology, Lake Placid, NY) was added for 2 hours. A fluorophore-labeled secondary antibody, Alexa Fluor 488 goat anti-rabbit IgG (Molecular Probes, Eugene, OR), was added for a 1 hour incubation protected from light.

### Detection of Secreted IRAP Protein

Secreted protein was detected with an enzyme-linked immunosorbent assay (ELISA) kit (R&D Systems, Minneapolis, MN). Isolated islets transfected with Ad-IRAP before transplantation and Ad-IRAP islet grafts that survived T-cell challenge for 90 days were cultured for 48 hours. The supernatant was then collected and assayed in triplicate for IRAP concentration by ELISA.

### Multiphoton Microscopy

Islets were imaged using a multiphoton laser scanning confocal microscope comprised of a titanium-sapphire ultrafast tunable laser system (Coherent Mira Model 900-F, Olympus Fluoview confocal scanning electronics), an Olympus IX70 inverted microscope with custom built power attenuation, and external PMT detection systems. The three-dimensional multiphoton data stack acquisition utilized 2 photon excitation at 800 nm (BFP) and 870 nm (Alexa Fluor 488, Cy 3), with fluorescence emission detected using D450/50M, HG510/50, and HQ580/60 steep bandpass emission filters (Chroma, Brattleboro, VT).

### Streptozotocin Treatment

NOD*scid* recipients were rendered diabetic with a single high dose (230 mg/kg) of streptozotocin (STZ) dissolved in a 0.9% physiological saline solution and immediately injected intraperitoneally [18]. The animals were monitored by blood glucose readings from the tail vein until hyperglycemia was established. An animal was considered diabetic after 2 consecutive readings of 300 mg/dL or higher.

### Islet Transplantation and Graft Recovery

A total of 300 to 400 islets were transplanted per mouse. Under sterile conditions, the recipient animal was anesthetized with a 2.5% solution of avertin, injected intraperitoneally at a dose of 0.015 to 0.017 mL/g body weight [19]. The kidney was externalized through a small incision in the flank. The islets were aspirated into PE50 tubing (Harvard Apparatus, Holliston, MA), centrifuged lightly, then deposited into the space between the capsule and the kidney. The kidney was replaced in the abdominal cavity and the incision closed with small wound clips. Either unmodified or transgene-bearing islets isolated from 3- to 4-week-old NOD mice were transplanted into a total of 92 STZ-treated NOD*scid* animals. Transplanted animals were monitored for 1 to 2 weeks to establish the primary function of the graft. Only animals exhibiting sustained euglycemia (70 to 150 mg/dL) during this time were included in the study. For graft recovery, the kidney was exposed as in the transplant procedure. The renal artery and vein were ligated with 6–0 silk suture and the kidney was separated from the body and retained for histologic analysis. The wound was closed as before and the animal allowed to recover. If hyperglycemia was evident in 2 to 5 days after graft removal, the animal was considered graft dependant.

### Autoimmune Challenge In Vivo

Spleens were removed from spontaneously diabetic NOD female mice (blood glucose $> 300$ mg/dL). A single-cell suspension was prepared by manual disruption of the spleen and passage through a 40-$\mu$m nylon mesh. NOD*scid* recipients to be challenged were injected intraperitoneally with approximately $20 \times 10^6$ spleen cells [20]. Blood glucose levels were monitored for 90 days. Any animal remaining euglycemic after 90 days had the graft-bearing kidney removed to determine if euglycemia was graft dependant.

### Statistical Analysis

A 2-sample $t$ test for independent samples with unequal variances was used to test for statistical significance of the mean time of graft function for each experimental animal group in comparison to the pooled control group. Kaplan-Meier analysis was used to calculate the survival curves.

### RESULTS

### Islet Transgene Infection Patterns

In our previous studies, NOD islets were infected ex vivo with 100 infectious particles per target cell (multiplicity of infection or MOI). Islets infected in this way and transplanted into a syngeneic host show transgene expression in excess of 100 days [3, 4]. For this study, we coinfected islet groups with 2 adenoviral vectors (100 MOI of each) for each gene combination. This was done so as not to dilute the infection rate for either transgene. Figure 1 is an example of islets genetically modified with 2 transgenic adenoviral vectors. The blue color (DAPI filter) is the result of cell infection with Ad-IDO-BFP, a vector containing IDO plus a blue fluorescent protein (BFP) in the construct. Unfortunately, this BFP was found to fade quickly. Therefore, this figure could not be used for a quantitative analysis of infection. The green color (fluorescene isothiocyanate [FITC] filter) represents cells infected with Ad-MnSOD. In this case, infected islets were stained with an MnSOD antibody followed by Alexa Fluor 488 goat anti-rabbit IgG (Molecular Probes, Eugene, OR), a fluorophore-labeled secondary antibody. Rhodamine phalloidin (red color) was used to label cellular F-actin as a counterstain. Most of the adeno-infected cells are found in the external layers of the islet as was also shown in previous studies [4]. The yellow color is a background effect that results from the superimposition of green and red fluorophores.

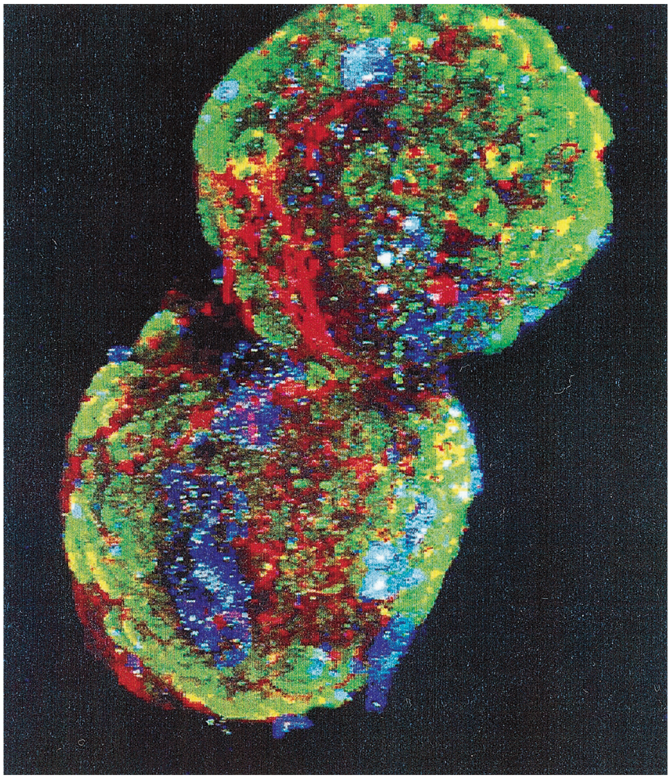

**FIGURE 1**
Distribution of transgene-bearing islet cells in whole murine islets after co-infection with 2 adenoviral transgene constructs. Islets were simultaneously incubated with 2 different constructs at 100 MOI each for 1 hour at RT with agitation. The blue color indicates the Ad-IDO-BFP construct. Green is produced by a FITC-labeled antibody bound to the Ad-MnSOD in the infected cells. The red color is a nuclear background stain. Yellow color is produced by the superimposition of green and red fluorophores. Original magnification 40×.

### Percentage of Islet Cells Infected by Each Viral Vector

After simultaneous adenoviral infection of 2 individual gene constructs into whole isolated NOD islets, FACS analysis was performed to identify the percentage of cells infected by each gene. Islets infected with Ad-IDO-BFP and Ad-MnSOD were disassociated into single cells 48 to 96 hours after infection and sorted to identify the percentage of cells carrying each marker gene. Figure 2*A* and *B* show representative dot plots of the results of 2 color FACS analysis. Figure 2*A* shows noninfected control islet cells gated so that the majority (>99%) are in the lower left (LL), double-negative quadrant. Figure 2*B* on the right shows the results of the combined gene constructs 96 hours after infection. Using the same gate as for controls, double-negative cells are in the LL quadrant (~70%), cells infected with Ad-IDO-BFP only are represented in the upper left (UL) quadrant (3.62%), Ad-MnSOD only are in the lower right (LR) quadrant (5.82%), and those co-infected with both vectors are in the upper right (UR) quadrant (14.32%). Adding these 3 quadrant values together for total cells infected, 23.76% of the islet cells were expressing either one or both transgenes in this group, with the majority (14.32%) coinfected with both vectors. The total number of cells expressing MnSOD is 20.14% (LR + UR) and those expressing IDO total 17.94% (UL + UR). The graph in Figure 2*C* shows the average percentage of single-gene and double-gene–expressing islet cells from 4 independently infected groups of islets. The last column represents an unrelated group of islets with a single-gene transferred, introduced here for comparison.

### Duration of Graft Function With Single-Gene Expression

Results from our 2 previous in vivo studies of Ad-IDO and Ad-MnSOD as single-gene modifications are summarized in Table 1 along with current results obtained from Ad-IRAP–expressing islet grafts [3, 4]. All three of these single genetic modifications resulted in significant prolongation of islet function when compared to controls. The NOD*scid* transplanted control mice listed in Table 1 include grafts composed of either

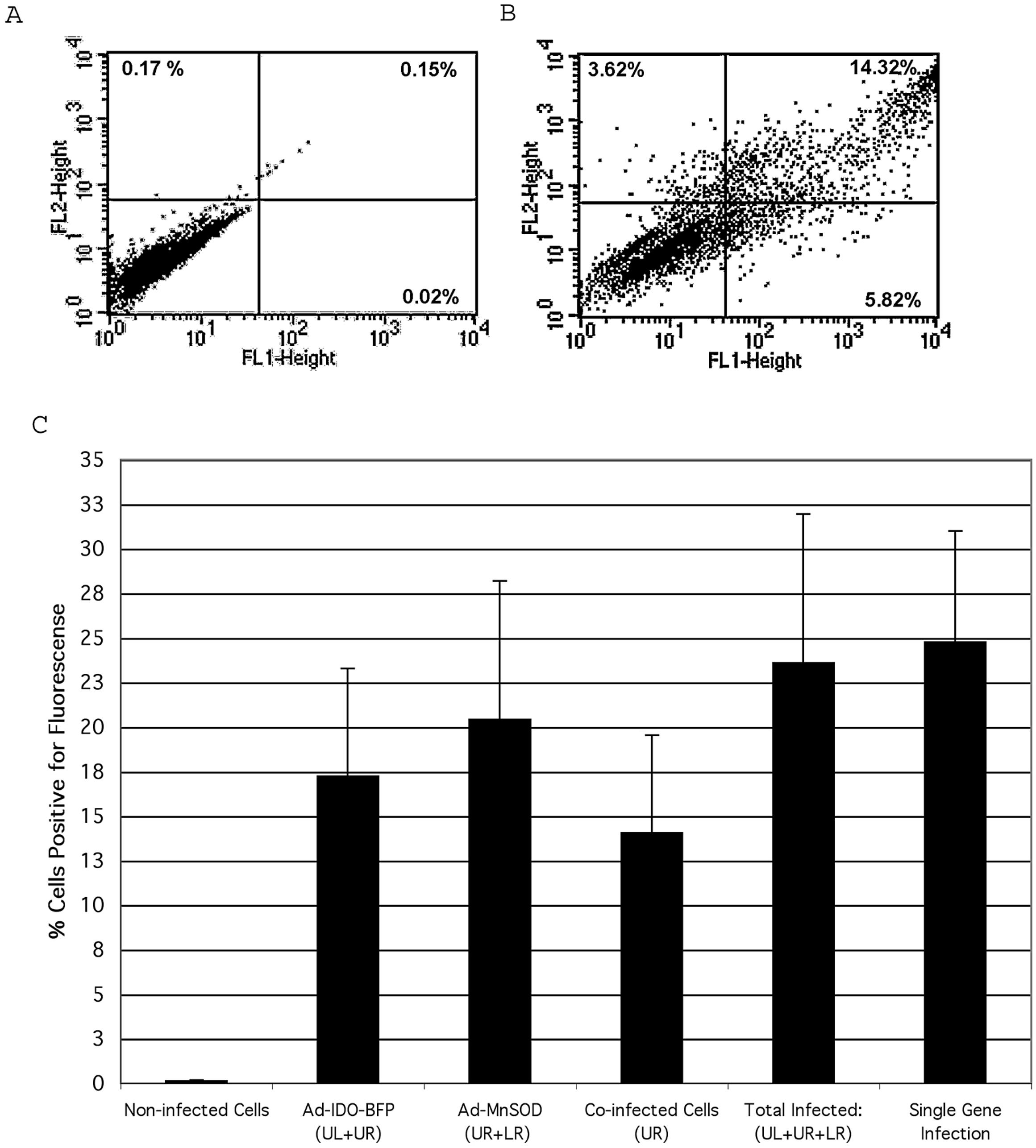

**FIGURE 2**

Quantitative analysis of gene transfer to islet cells. Islets simultaneously infected with Ad-IDO-BFP plus Ad-MnSOD were cultured for 4 days and dispersed to single cells for FACS analysis. (*A*) Representative dot plot of unmodified (control) islet cells. (*B*) Representative dot plot of infected cells. Lower left (LL) quadrant represents double-negative cells, upper left (UL) quadrant represents cells expressing IDO only, lower right (LR) represents cells expressing MnSOD only, and upper right (UR) represents cells coinfected with both IDO and MnSOD. (*C*) Results of FACS analysis from 4 independent experiments calculated as mean percentage ± SEM of islet cells transfected with adeno-gene constructs. The first column on the left represents noninfected control cells. The central columns divide the combination infections into 4 categories: the percentage of total cells bearing Ad-IDO-BFP (second column), the total cells bearing Ad-MnSOD (third column), cells that coexpress Ad-IDO-BFP and Ad-MnSOD (fourth column), and the total amount of infected cells in the islet population (fifth column). The last column on the right represents an unrelated single gene infection shown here for comparison.

**TABLE 1**
Duration of function of in vivo T cell–challenged islet grafts

| | *n* | Mean ± SD | Range | *P* value |
|---|---|---|---|---|
| Single gene modifications* | | | | |
| Ad-IDO | 14 | 43 ± 15 | 24–83 | <.005 |
| Ad-MnSOD | 12 | 47 ± 14 | 32–76 | <.005 |
| Ad-IRAP | 11 | 46 ± 23 | 16–90 | .005 |
| Combinations* | | | | |
| Ad-IDO + Ad-MnSOD | 11 | 41 ± 22 | 19–77 | .062 |
| Ad-MnSOD + Ad-IRAP | 9 | 50 ± 18 | 30–90 | <.005 |
| Ad-IDO + Ad-IRAP | 10 | 41 ± 20 | 19–90 | .066 |
| Controls | | | | |
| Unmodified | 11 | 31 ± 9 | 19–61 | |
| Ad-LacZ* | 14 | 28 ± 14 | 7–61 | |
| Non-Tx | 18 | 33 ± 7 | 23–49 | |
| Pooled** | 43 | 32 ± 11 | 7–61 | |

*Note.* Length of function of modified islet grafts was measured as days of euglycemia after diabetogenic spleen cell challenge. For each group, day 0 was considered the day of challenge; "n" is the number of animals per group; mean ± SD is the mean number plus/minus standard deviation of days of euglycemia after challenge. Range is the shortest and longest duration of eulycemia of individuals within each group. *P* value was calculated against the pooled controls.
*Islets were incubated with a concentration of 100 MOI of each gene per infection.
**Unmodified, Ad-LacZ, and Non-Tx control groups were pooled together for statistical calculations.

**TABLE 2**
Secreted IRAP production (ng/mL)

| | Unmodified | Ad-LacZ | Ad-IRAP |
|---|---|---|---|
| Before transplantation | Undetectable | Undetectable | 29 ± 3.3 |
| After transplantation | Undetectable | Undetectable | 8.2 ± 5.1 |

*Note.* Production of IRAP by murine islets after infection with Ad-IRAP (100 MOI) and AD-LacZ (100 MOI) or unmodified islets. Before transplantation: groups of 300 to 400 islets were isolated, exposed to adenoviral vectors for 1 hour or not (unmodified group) and incubated at 37°C for 48 hours before the supernatant was tested by ELISA in triplicate. After transplantation: If still functional 90 days after T-cell challenge, the graft was removed and placed in culture for 48 hours. These included one graft each of IRAP alone, MnSOD & IRAP, and IDO & IRAP. The supernatant was assayed as before. For this experiment, unmodified and Ad-LacZ grafts were not challenged.

unmodified islets or Ad-LacZ–infected islets from young NOD mice. A third group of nontransplanted NOD*scid* mice was included with every group of spleen cell challenges as a control for the adoptive transfer of disease. A survival curve of the 3 control groups is shown in the bottom panel of Figure 3. For purposes of calculating statistical significance, these 3 control groups were pooled together.

For the single-gene–bearing grafts, it was previously found that both Ad-IDO and Ad-MnSOD significantly prolonged islet graft function, with strong statistical significance for mean days of function after challenge compared to the pooled control group ($P < .005$). In the new data obtained in this study, Ad-IRAP was also found to significantly lengthen graft function. The middle graph in Figure 3 tracks the length of function (in days) after T-cell challenge for each set of grafts, with single-gene expression compared to the pooled controls. The single-gene group containing Ad-IRAP had the only animal that remained euglycemic for the full 90-day goal of the experiment. The graft-bearing kidney was removed from the animal and the graft was cultured in 2 mL medium and incubated at 37°C for 48 hours. The supernatant showed that IRAP was still detectable by ELISA whereas levels in control grafts were undetectable (Table 2).

### Duration of Graft Function With Double-Gene Expression

In order to test for possible synergistic effects of multiple genes, islets were simultaneously infected with 2 transgene constructs. The results of the in vivo experiments are summarized in Table 1. Although the IDO plus MnSOD–expressing islets showed a strong trend toward increased survival, with a mean time of graft function of 41 days, these results did not quite reach statistical significance when compared to the pooled control group ($P = .062$). The case was similar for IRAP and IDO, also with a mean of 41 days ($P = .066$). The combination of IRAP and MnSOD however, was significantly longer at 50 days than the pooled controls at 32 days ($P \leq .005$). As in the case of the single-gene groups, only the double-gene groups containing IRAP had animals that remained euglycemic for 90 days (one in each group). These grafts were removed and assayed in triplicate for secreted IRAP by ELISA. A mean average of the grafts plus the single-gene graft containing IRAP showed that the grafts were secreting about a third of that before transplant (Table 2). The top graph in Figure 3 tracks the length of function (in days) after challenge for the 3 sets of grafts expressing transgene combinations compared to the pooled controls. Figure 4 shows the individual glycemic profiles for the combination groups and randomly selected controls.

### DISCUSSION

We have shown in islet cells that it is possible to simultaneously infect with 2 adenoviral vectors, each expressing a different gene, and that the total percentage of infected islet cells is similar, on average, to islets infected with a single vector. We

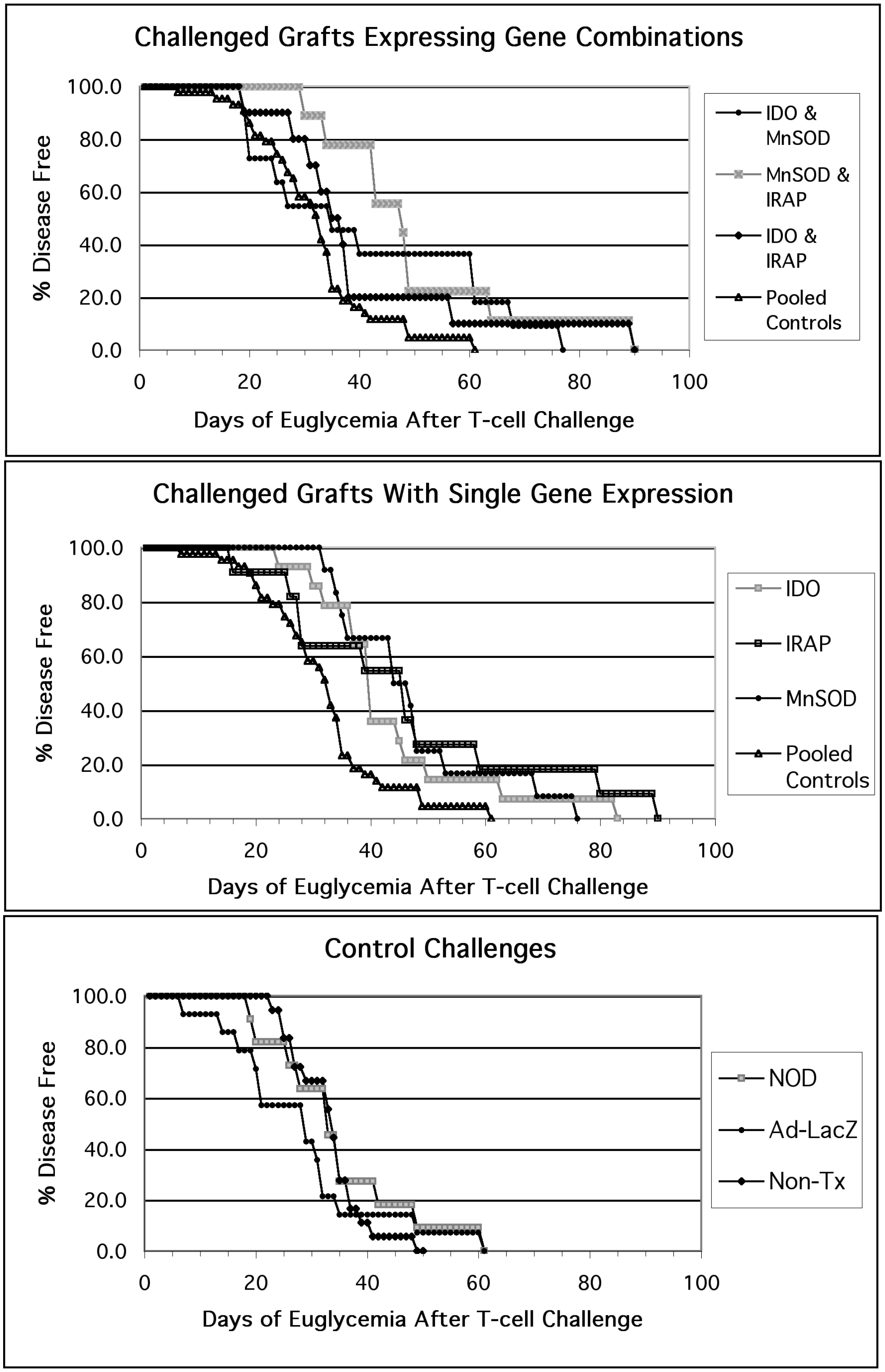

**FIGURE 3**

Survival data of islet grafts after challenge with spleen cells from spontaneously diabetic NOD mice. The top panel shows islet grafts expressing gene combinations. IDO and IRAP (♦), IDO and MnSOD (●), IRAP and MnSOD (×), and pooled controls (Δ). Middle panel shows single gene islet grafts with either IDO (□), IRAP (■), or MnSOD (●) gene expression and pooled controls (Δ). The bottom panel shows the control groups consisting of unmodified NOD islet grafts (□), Ad-LacZ expressing NOD islet grafts (●), and nontransplanted (Non-Tx) NOD*scid* mice (♦) included as controls for adoptive transfer of disease.

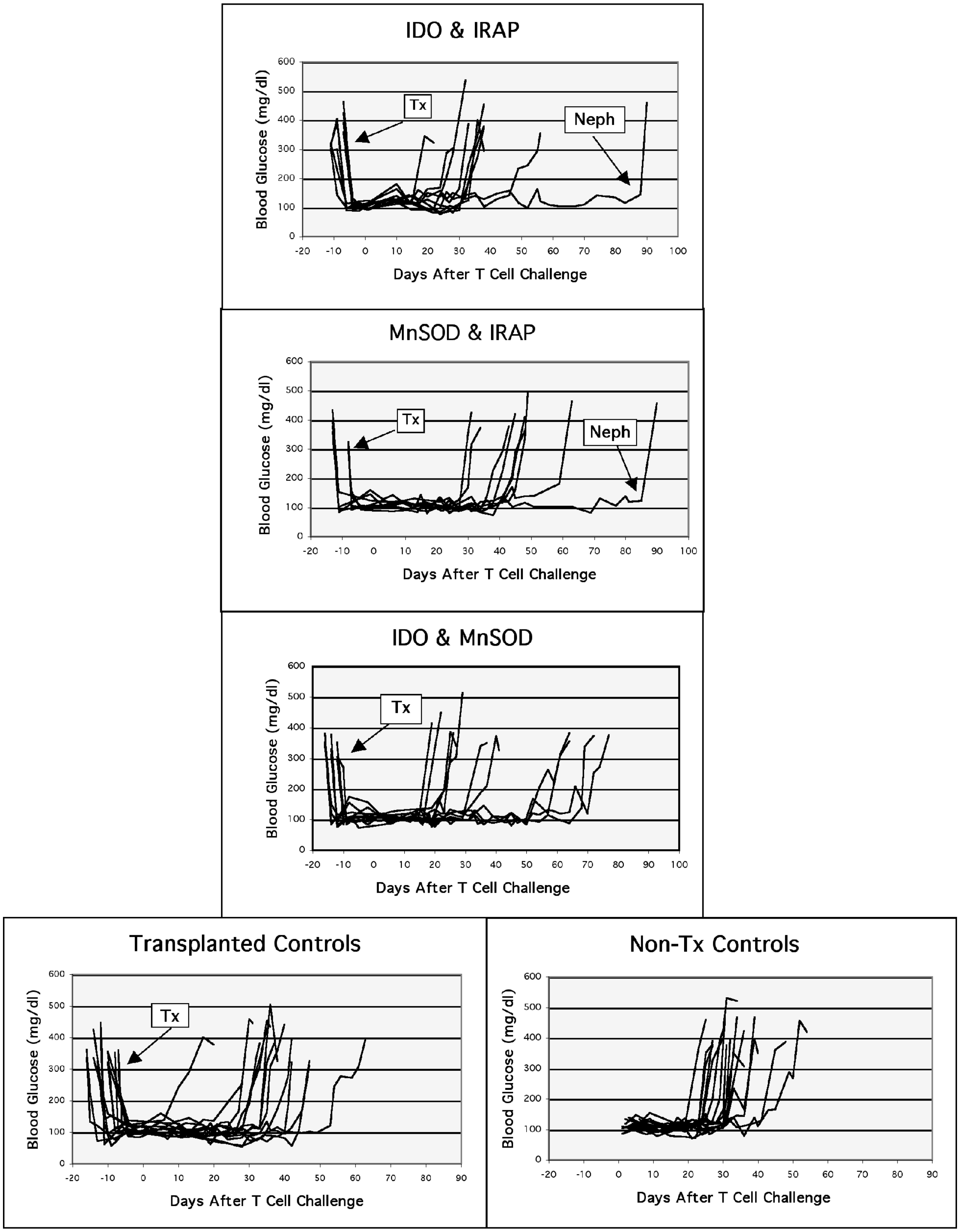

**FIGURE 4**

Individual glycemic profiles of combination islet grafts and controls. The day of T-cell challenge is indicated as day 0. Other points indicated include day of transplantation (Tx) and day of graft-bearing kidney removal (Neph). Glycemic values of >300 mg/dL were considered as confirmation of diabetes. Panels in order from top are IDO & IRAP, MnSOD & IRAP, IDO & MnSOD, and controls at the bottom. The controls were split into 2 groups for clarity, transplanted controls on the lower left and Non-Tx (nontransplanted controls) on the lower right. Of the 25 unmodified and Ad-LacZ control transplants actually performed, half were selected at random for editorial purposes, and shown in this figure. Individuals of the other groups are shown in their entirety. The nontransplanted animals were controls for the transfer of disease and were included in each T-cell–challenged group of transplanted animals to prove the efficiency of the diabetic splenocytes.

have also shown by FACS analysis that 2 genes may colocalize in the same islet cell and that the majority of infected cells express both transgenes. This expression does not appear to interfere with graft function in vivo. The blood glucose levels of diabetic animals normalize at comparable rates whether given modified or control islet grafts (Figure 4).

Our in vivo data show that transplanted islets overexpressing IRAP alone functioned longer than MnSOD and IDO and significantly longer than controls after exposure to diabetogenic T-cells. Therefore, we included it in the combination trials to determine if there was any indication of a positive synergistic effect. Our reasons for coupling IRAP with IDO were based in part on observations from previous studies such as those from Munn and colleagues [6], who put forth the idea that IDO expression in the environment of antigen-presenting cells may be able not only to inhibit T-cell proliferation, but also to induce T-cell death. They found that T-cells activated under tryptophan-deficient conditions arrest midway through the G1 stage of the cell cycle and become susceptible to apoptosis. Arresting T-cell activation at the mid-G1 stage is also the way some immunosuppressive drugs work, including rapamycin and leflunomide [21, 22]. Therefore, combining IDO with IRAP could then provide an additional dimension to islet protection by blocking both T-cell activation and IL-1$\beta$ receptor binding on the target cell.

Other recent studies have revealed that although MnSOD is a potent antioxidant enzyme, its activity is thought to be defeated over time because of increased NO levels. This is believed because the reaction of $O_2^-$ combining with NO to produce peroxynitrite ($OONO^-$) is faster than the $O_2^-$ dismutation reaction of MnSOD [23]. The resultant cytotoxic radical, peroxynitrite, accumulates to a concentration that effectively quenches MnSOD activity, thereby allowing oxygen radicals to damage tissue. IRAP expression works to prevent NO from being generated in response to increased IL-1$\beta$ levels [24]. With less NO to form peroxynitrite, the length of time MnSOD is active should increase and therefore the length of time the islets function in grafts overexpressing MnSOD and IRAP should also increase beyond those overexpressing MnSOD alone.

The model used in our study tests a therapeutic gene's ability to protect islets against the destructive power of a full-blown autoimmune attack. The results show that these gene combinations could at most protect the islet grafts for the same length of time as could the individual genes. The mean length of graft function for islets bearing the combinations of IDO and IRAP ($41 \pm 20$ days), MnSOD and IRAP ($50 \pm 18$ days), or MnSOD and IDO ($41 \pm 22$) was not statistically different compared to the length of graft function of IDO alone ($43 \pm 15$ days), MnSOD alone ($47 \pm 14$), or IRAP alone ($46 \pm 23$).

There are several possible explanations for our results. The lack of synergistic action of these genes could be due to different subpopulations of autoreactive T-cells acting through different pathways. Each subset can only be blocked for a certain amount of time before the obstacle is overcome. The combination of genes examined here seems not to enhance the blockade of all T-cell subpopulations. Instead, they offer a limited blocking, each directed to a specific T-cell–mediated mechanism. Another explanation is that autoreactive T-cells quickly learn how to adapt their receptors and biochemical activities so as to be only temporarily limited by the immunomodulatory genetic expression transferred into the target islets. Additionally, the protected factors generated by the transgenic proteins may be produced/released in decreasing amounts with time, eventually allowing the T-cells to supercede. One last possibility is that because there was less protein detected in the supernatant of the IRAP grafts after 90 days (Table 2), some of the Ad-IRAP–infected islet cells either stopped secreting the protein or died/were killed and therefore IRAP production was lost.

The reasons we had for choosing these antioxidant and immunoregulatory genes were sound. Previous in vitro testing proved that these genes were present in the target cells after adenoviral infection and both the islet cells and the genes functioned normally and as expected. The gene proteins were produced by the target cells and detected in the medium supernatant. Nevertheless, the dynamics of a functioning complex immune system in vivo is still not entirely understood. We were able to significantly prolong islet function with overexpression of IRAP, IDO, and MnSOD gene proteins but a combination of these genes did not work in synergy to extend graft function further than any gene individually.

In conclusion, even though these specific genes did not provide complete protection to donor islets, the method of manipulating islets ex vivo with therapeutic genes has proven to be of value in extending the functional life of transplanted islets in this mouse model of autoimmune diabetes. It is an excellent system to test other candidate genes to determine in vivo therapeutic ability and does not preclude being used in conjunction with other therapies aimed at curing this complex disease.

### REFERENCES

[1] Shapiro, A. M., Lakey, J. R., Ryan, E. A., Korbutt, G. S., Toth, E., Warnock, G. L., Kneteman, N. M., and Rajotte, R. V. (2000) Islet transplantation in seven patients with type 1 diabetes mellitus using a glucocorticoid-free immunosuppressive regimen. *N. Engl. J. Med.,* **343,** 230–238.

[2] Olyaei, A. J., de Mattos, A. M., and Bennett, W. M. (2001) Nephrotoxicity of immunosuppressive drugs: New insight and preventive strategies. *Curr. Opin. Crit. Care,* **7,** 384–389.

[3] Alexander, A. M., Crawford, M., Bertera, S., Rudert, W. A., Takikawa, O., Robbins, P. D., and Trucco, M. (2002) Indoleamine 2,3-dioxygenase expression in transplanted NOD Islets prolongs graft survival after adoptive transfer of diabetogenic splenocytes. *Diabetes,* **51,** 356–365.

[4] Bertera, S., Crawford, M. L., Alexander, A. M., Papworth, G. D., Watkins, S. C., Robbins, P. D., and Trucco, M. (2003) Gene transfer of manganese superoxide dismutase extends islet graft function in a mouse model of autoimmune diabetes. *Diabetes,* **52,** 387–393.

[5] Giannoukakis N., Rudert, W. A., Ghivizzani, S. C., Gambotto, A., Ricordi, C., Trucco, M., and Robbins, P. D. (1999) Adenoviral gene transfer of the interleukin-1 receptor antagonist protein to human islets prevents IL-1beta-induced beta-cell impairment and activation of islet cell apoptosis in vitro. *Diabetes,* **48,** 1730–1736.

[6] Munn, D. H., Shafizadeh, E., Attwood, J. T., Bondarev, I., Pashine, A., and Mellor, A. L. (1999) Inhibition of T cell proliferation by macrophage tryptophan catabolism. *J. Exp. Med.,* **189,** 1363–1372.

[7] Mellor, A. L., Keskin, D. B., Johnson, T., Chandler, P., and Munn, D. H. (2002) Cells expressing indoleamine 2,3-dioxygenase inhibit T cell responses. *J. Immunol.,* **168,** 3771–3776.

[8] Evans, C. H., and Robbins, P. D. (1994) The interleukin-1 receptor antagonist and its delivery by gene transfer. *Receptor,* **4,** 9–15.

[9] Rabinovitch, A., Suarez-Pinzon, W. L., Sorensen, O., and Bleackley, R. C. (1996) Inducible nitric oxide synthase (iNOS) in pancreatic islets of nonobese diabetic mice: Identification of iNOS-expressing cells and relationships to cytokines expressed in the islets. *Endocrinology,* **137,** 2093–2099.

[10] Yamada, K., Takane-Gyotoku, N., Yuan, X., Ichikawa, F., Inada, C, and Nonaka, K. (1996) Mouse islet cell lysis mediated by interleukin-1-induced Fas. *Diabetologia,* **39,** 1306–1312.

[11] Lenzen, S., Drinkgern, J., and Tiedge, M. (1996) Low antioxidant enzyme gene expression in pancreatic islets compared with various other mouse tissues. *Free Radic. Biol. & Med.,* **20,** 463–466.

[12] Liu, R., Buettner, G. R., and Oberley, L. W. (2000) Oxygen free radicals mediate the induction of manganese superoxide dismutase gene expression by TNF-alpha. *Free Radic. Biol. & Med.,* **28,** 1197–1205.

[13] Strandell, E., Buschard, K., Saldeen, J., and Welsh, N. (1995) Interleukin-1 beta induces the expression of hsp70, heme oxygenase and Mn-SOD in FACS-purified rat islet beta-cells, but not in alpha-cells. *Immunol. Lett.,* **48,** 145–148.

[14] Grankvist, K., Marklund, S. L., and Taljedal, I. B. (1981) CuZn-superoxide dismutase, Mn-superoxide dismutase, catalase and glutathione peroxidase in pancreatic islets and other tissues in the mouse. *Biochem. J.,* **199,** 393–398.

[15] Gotoh, M., Maki, T., Kiyoizumi, T., Satomi, S., and Monaco, A. P. (1985) An improved method for isolation of mouse pancreatic islets. *Transplantation,* **40,** 437–438.

[16] Giannoukakis, N., Thomson, A., and Robbins, P. (1999) Gene therapy in transplantation [Review]. *Gene Therapy,* **6,** 1499–1511.

[17] Muruve, D. A., Manfro, R. C., Strom, T. B., and Libermann, T. A. (1997) Ex vivo adenovirus-mediated gene delivery leads to long-term expression in pancreatic islet transplants. *Transplantation,* **64,** 542–546.

[18] Like, A. A., Appel, M. C., Williams, R. M., and Rossini, A. A. (1978) Streptozotocin-induced pancreatic insulitis in mice. Morphologic and physiologic studies. *Lab. Invest.,* **38,** 470–486.

[19] Hogan, B., Beddington, R., Costantini, F., and Lacy, E. (1994) Avertin, Anesthetic *Manipulating the Mouse Embryo, A Laborotory Manual,* pp. 416. Cold Spring Harbor, NY, Cold Spring Harbor Laborotory Press.

[20] Gerling, I. C., Friedman, H., Greiner, D. L., Shultz, L. D., and Leiter, E. H. (1994) Multiple low-dose streptozocin-induced diabetes in NOD-scid/scid mice in the absence of functional lymphocytes. *Diabetes,* **43,** 433–440.

[21] Terada, N., Takase, K., Papst, P., Nairn, A. C., and Gelfand, E. W. (1995) Rapamycin inhibits ribosomal protein synthesis and induces G1 prolongation in mitogen-activated T lymphocytes. *J. Immunol.,* **155,** 3418–3426.

[22] Cherwinski, H. M., Cohn, R. G., Cheung, P., Webster, D. J., Xu, Y. Z., Caulfield, J. P., Young, J. M., Nakano, G., and Ransom, J. T. (1995) The immunosuppressant leflunomide inhibits lymphocyte proliferation by inhibiting pyrimidine biosynthesis. *J. Pharmacol. Exp. Ther.,* **275,** 1043–1049.

[23] Quijano, C., Hernandez-Saavedra, D., Castro, L., McCord, J. M., Freeman, B. A., and Radi, R. (2001) Reaction of peroxynitrite with Mn-superoxide dismutase. Role of the metal center in decomposition kinetics and nitration. *J. Biol. Chem.,* **276,** 11631–11638.

[24] MacMillan-Crow, L. A., Crow, J. P., and Thompson, J. A. (1998) Peroxynitrite-mediated inactivation of manganese superoxide dismutase involves nitration and oxidation of critical tyrosine residues. *Biochemistry,* **37,** 1613–1622.

